# Editorial: Basic and applied research toward the development of vaccines against African swine fever virus

**DOI:** 10.3389/fimmu.2025.1658984

**Published:** 2025-08-14

**Authors:** Daniel Pérez-Núñez, Sandra Blome, Linda K. Dixon

**Affiliations:** ^1^ Microbes in Health and Welfare Department, Centro de Biología Molecular-Consejo Superior Investigaciones Científicas Severo Ochoa (CBM-CSIC), Madrid, Spain; ^2^ Friedrich-Loeffler-Institut, Federal Research Institute for Animal Health, Institute of Diagnostic Virology, Greifswald-Isle of Riems, Germany; ^3^ The Pirbright Institute, Woking, United Kingdom

**Keywords:** ASFV, vaccines, virulence factors, innate immunity, IFN-I, c-GAS/STING, tropism, cell line

African swine fever virus (ASFV) is a complex dsDNA virus that causes a disease that is frequently fatal, ASF. This has a high socio-economic impact. ASFV causes acute disease in both domestic pigs and wild boar. Originating in sub-Saharan Africa, it causes inapparent infections in African wild suids, including warthogs, and in soft tick vectors of the virus. Its rapid spread across Europe, Asia, Oceania, and the Caribbean highlights the urgent need for effective vaccines.

Currently, live attenuated vaccines (LAVs) represent the most effective approach, though these have limited availability and possible safety concerns. For ASF-free regions, developing alternative approaches such as vector, subunit, or replication-deficient vaccines may be more appropriate, but this requires greater knowledge of the mechanisms and antigens that are important for protection. Factors limiting vaccine development include the large number of proteins encoded, limited knowledge of the virus replication and immune evasion, factors influencing host and cellular tropism, efficient cell lines for the growth of vaccine prototypes, and poor understanding of mechanisms and correlates of pathogenesis and protection in pigs.

This Research Topic, titled “*Basic and Applied Research Toward the Development of Vaccines Against African Swine Fever Virus*,” aims to highlight important developments in this critical area of research, from pathogenesis and immune response to functional topics such as vaccination and diagnostic tools and strategies.

The ASFV genome contains up to ~190 open reading frames (ORFs) based on DNA sequence analysis. These are transcribed into mRNAs in the cytoplasm by the virus-encoded RNA polymerase and stage-specific transcription factors (1). Cackett et al. present the first accurate map of the transcripts across the ASFV genome. This was achieved by determining full-length sequences of viral mRNAs at early and late stages of infection in combination with the identification of transcript 5’ start and 3’ termination sites. The study also identified consensus early and late ASFV promoter motifs and termination sequences. The transcript read through to downstream ORFs was identified particularly late during infection. This information is critical to confirming which ORFs are transcribed and to accurately predict the sequences of proteins encoded and the timing of their expression. The information also aids in the design of genome deletions and modifications used in the construction of LAVs.

To improve vaccine development, better understanding of the factors that regulate the immune responses leading to protection against virulent virus challenge is required. Radulovic et al. have extended their previous study, which compared responses of specific pathogen-free (SPF) pigs with those of farm pigs following inoculation with a moderately virulent ASFV strain (2). The SPF and farm pigs differed in their gut microbiome and their basal immune activation status. Less severe disease was observed in SPF pigs than in conventional farm pigs. In this article, responses to the challenge after 4 months with a virulent virus were compared. The SPF pigs were fully protected and showed little or no viremia. In contrast, farm pigs developed high viremia, proinflammatory cytokine responses, and severe clinical signs. Around 40% of the farm pigs reached the humane endpoint. These striking results indicate that limited prior immune exposure to other pathogens and/or the microbiome composition of SPF pigs promotes resilience to infection with a moderately virulent strain and the development of strong protective immunity against virulent ASFV challenge (Radulovic et al.).

The limited availability of ASFV-susceptible, continuously growing cell lines representing the natural target cells, monocytes/macrophages, has hindered research and vaccine development. Takenouchi et al. describe an immortalised macrophage cell line from red river hogs (*Potamochoerus porcus*), African natural hosts that tolerate ASFV infection. The RRH cell line was confirmed to have a macrophage-like phenotype by analysis of cell surface markers, activation of pro-inflammatory cytokines and IFN-β production, and phagocytic activity. Interestingly, the replication kinetics of ASFV in the RRH cell line were more variable and reached lower titres compared with the porcine macrophage-derived cell line, IPKM. These cells provide an excellent new tool to investigate ASFV replication and elucidate the innate immune responses in naturally tolerant host species. In addition, these cells would enable large-scale vaccine production and standardised viral growth assays, eliminating the need for primary pig cells. This tool is critical for current and future vaccine platforms such as LAVs.

The development of ASFV subunit vaccines is hindered by a lack of knowledge of protective antigens and an effective delivery system. Gao et al. investigate recombinant *Saccaromyces cerevisiae* (SC), which has been certified by the US Food and Drug Administration as safe for use in the food industry, as a novel method to deliver ASFV antigens. In this proof of concept study, sequences, which code for the antigenic regions from 8 ASFV proteins fused to a Dendritic Cell targeting peptide, were inserted into yeast chromosomes. The ASFV proteins were stably expressed on the surface of recombinant SC strains. Oral immunisation of mice induced strong humoral, mucosal, and cellular Th1 and Th2 immune responses. Future testing in swine, including challenge studies, will determine the vaccine potential of this novel approach for ASFV.

The field evaluation of live attenuated vaccines requires sensitive methods to detect virus replication and ideally a method that can distinguish infected animals from vaccinated animals (DIVA). Luan et al. describe a sensitive visual assay for detecting ASFV genomes. This was developed using the KP177R gene as a target since this gene, although well conserved, can be deleted from the genome without affecting virus replication or virulence in pigs. Thus, KP177R represents a possible target gene to be deleted from LAVs. The detection method first generated amplicons using recombinant polymerase amplification. Subsequently, these were recognised by a guide RNA to activate the trans-cleavage activity of Cas12a protein, thereby leading to non-specific cleavage of single-stranded DNA as well as a corresponding colour reaction. The detection assay had a limit of detection of 6.8 genome copies/mL, and was completed in 30 min. The assay could be used in the field on farms.
